# Clinical and laboratory factors associated with splenic dysfunction among patients with sickle cell disease in a malaria endemic region

**DOI:** 10.1093/trstmh/trad059

**Published:** 2023-08-24

**Authors:** Adama I Ladu, Ngamarju A Satumari, Aisha M Abba, Fatima A Abulfathi, Caroline Jeffery, Adekunle Adekile, Imelda Bates

**Affiliations:** Department of International Public Health, Liverpool School of Tropical Medicine, Liverpool, L3 5QA, UK; Department of Haematology, Faculty of Basic Clinical Sciences, University of Maiduguri, Maiduguri, Borno State 60001, Nigeria; Department of Medical Laboratory Science, Faculty of Allied Health Sciences, University of Maiduguri, Maiduguri, Borno State 60001, Nigeria; Department of Haematology, Faculty of Basic Clinical Sciences, University of Maiduguri, Maiduguri, Borno, 60001, Nigeria; Department of Haematology, Faculty of Basic Clinical Sciences, University of Maiduguri, Maiduguri, Borno, 60001, Nigeria; Department of International Public Health, Liverpool School of Tropical Medicine, Liverpool, L3 5QA, UK; Department of Clinical Infection, Microbiology and Immunology, Faculty of Health and Life Sciences, University of Liverpool, Liverpool, L69 3BX, UK; Department of Paediatrics, Faculty of Medicine, Kuwait University, Saffat 13110, Kuwait; Department of International Public Health, Liverpool School of Tropical Medicine, Liverpool, L3 5QA, UK

**Keywords:** Hb F, Howell–Jolly bodies, Red cell inclusions, Sickle cell disease, Spleen

## Abstract

**Background:**

Although loss of splenic function is the expected natural course for individuals with sickle cell disease (SCD), factors such as high HbF and coexistence of alpha thalassemia may ameliorate this process. We evaluated factors associated with two surrogate markers of spleen dysfunction, namely Howell–Jolly bodies (HJBs) and argyrophilic inclusion (AI) red cell counts, among patients with SCD.

**Methods:**

Cross-sectional data of 182 patients with SCD (median age 11 y; 1–45 y) and 102 normal controls (median age 12 y; 1–32 y) were evaluated. Blood tests including full blood count, serum chemistry and high-performance liquid chromatography were performed. The HJB and AI red cell counts were performed on peripheral blood smears.

**Results:**

The percentages of HJB and AI red cells rose significantly with increasing age in the SCD group. On regression analysis, the frequency of HJB red cells associated positively with mean corpuscular hemoglobin (MCH) (β=0.289; p=0.001) and negatively with HbF (β=−0.259; p=0.002). The AI red cell counts also associated positively with MCH (β=0.321; *P*=0.001) and negatively with HbF (β=−0.242; p=0.020).

**Conclusions:**

Data from this study indicate that the negative association of HbF with both markers of splenic dysfunction among our patients with SCD residing in a malaria endemic region is similar to findings elsewhere of its ameliorating effect on splenic dysfunction.

## Introduction

The spleen plays an important role in the defense against infections and is one of the earliest organs to be affected in sickle cell disease (SCD). This occurs as a result of repeated cycles of ischemia and infarction within the organ.^1^ Observations among patients with SCD residing in the Western hemisphere indicate that splenic dysfunction starts as early as 6 months of age and the majority of patients are affected by 2 y of life.^2–4^ The experience is, however, different in patients with SCD in Africa, Asia and the Middle East, where splenic dysfunction starts much later.^5–9^ The age variability at which splenic dysfunction starts, the rate of progression and consequently the stage at which it becomes clinically significant can be related to several factors including the coexistence of alpha thalassemia, high level of HbF and malaria infection.^7,8,10^ Supportive therapies including the use of hydroxyurea (HU), chronic red cell transfusions and stem cell therapy have also been associated with reversal or prolongation of spleen function among patients with SCD.^10–13^ The widespread use of these therapies may therefore influence the rate of splenic dysfunction among patients.

Given the role the spleen plays in protection against infections, splenic dysfunction increases vulnerability to invasive infections with encapsulated bacteria and parasitic infections^14,15^; however, the prevalence of splenic dysfunction and factors associated with its development are largely unknown in sub-Saharan Africa, the region where the majority of patients with SCD reside.^16^ This is because most of the tests used to assess spleen function, such as radionuclide scans and percentage of pitted red cells using contrast enhancing microscopy, are not readily available in most African countries. Thus, factors associated with splenic dysfunction among patients with SCD in Africa have not yet been fully investigated. We recently employed the presence of two red cell containing inclusions, namely Howell–Jolly bodies (HJBs) and argyrophilic (silver staining) inclusion (AI) red cells, to assess splenic dysfunction among our patients with SCD.^17^ In the present study, we aimed to investigate the variation in both markers of splenic dysfunction (i.e. HJB and AI) with age among the patients with SCD and compare results with those of healthy controls. Also, the relationship between the markers of splenic dysfunction with clinical outcomes and laboratory variables among the patients with SCD were explored.

## Methods

### Study design and participants

This was a prospective cross-sectional study performed over 14 months (October 2020 to November 2021). The study participants consisted of steady state patients with SCD (children and adults). The patients were recruited consecutively when they presented for their routine appointment at the pediatric and adult hematology out-patient clinics of the University of Maiduguri Teaching Hospital, North-Eastern Nigeria. Healthy individuals comprising medical students, children of hospital personnel and pediatric patients on post-op follow-up in the surgical clinic (without any acute or chronic illness likely to affect splenic function) served as the control population. The study participants were divided into four age groups: 1. 1–4 y; 2. 5–9 y; 3. 10–14 y; and 4. 15 y and above. Other aspects of this cohort have previously been studied.^18^

### Routine clinical data and laboratory assessment

At enrolment, a case report form was used to obtain demographic characteristics and self-reported medical history from the patients (or their carers) including the frequency of hospitalisation, febrile episodes and painful crises over the preceding 12 months. The history of current HU therapy and history of total lifetime transfusion received were also documented. Whole peripheral blood was collected from all the patients into plain and ethylenediamine tetra-acetic acid (EDTA)-containing tubes. Full blood counts were performed with the use of an automated analyser (Siemens). Reticulocytes were counted using the standard method with new methylene blue staining.^19^ Biochemical tests were performed using a chemical analyser (Hitachi Cobas C311, Roche Instrument Centre, Rotkreuz, Switzerland). Hemoglobin phenotype was performed by using ion-exchange high-performance liquid chromatography (HPLC) on an automatic analyser (Bio-Rad, Hercules, CA, USA).

Splenic function was assessed by using a manual estimation of HJBs and AI containing red cells as previously described.^17^ Blood smears for HJB red cells were fixed for 1 min in methanol and stained using the classical May–Grunwald Giemsa stain procedure. Blood smears for identification of AI red cells were fixed for 3 min in formalin and stained for 20 min at 38°C in dark using the silver stain. A minimum of 500 consecutive red cells per blood film for the AI count and 400 red cells for the HJB count were examined for the presence of the respective inclusion. The AI and HJB counts were expressed as percentages of the total red cells counted. Patients who had received blood transfusion over the last 3 months were excluded, as this would have interfered with the accuracy of the HPLC result and the parameters of the blood smears for spleen function.

### Statistical analysis

The data were analysed using Statistical Package for the Social Sciences (SPSS) (version 25; SPSS, Chicago, IL, USA). Categorical data were summarised using frequencies and proportions, while continuous data were summarised using descriptive statistics. Comparisons of HJB and AI red cell counts across the age groups of patients with SCD and controls were performed using the Mann–Whitney U test. Factors potentially associated with both markers of splenic dysfunction were analysed individually using univariate regression analysis. The goal of the analysis was to identify clinical and laboratory factors associated with increased levels of both markers. The presence of collinearity among the independent factors was explored and all variables with a variance inflation factor of >10 were excluded. A full model analysis containing all the significant variables was performed using the backward elimination method to evaluate the independent effects of each covariate by controlling the effects of other variables. The adjusted odds ratios and 95% CIs were computed. A p-value of <0.05 was considered statistically significant.

## Results

### Clinical characteristics of study participants

The study comprised 182 patients with SCD (median age 11 y; range 1–45 y) and 102 controls (median age 12 y; range 1–32 y). The Hb phenotypes of the patients with SCD consisted of 175 homozygous sickle cell disease (HbSS) (96.2%), five sickle-hemoglobin C disease (HbSC) (2.7%) and two sickle cell β-thalassemia (HbSβ) (1.1%). The majority of the patients with SCD reported one or more episodes of fever (89%) and painful crises (77%) over the last 12 months, and 63 (34.6%) patients required in-patient hospitalisation. The majority of parents and guardians of the younger patients admitted having completed routine childhood immunisation for their children, although most of the older patients with SCD were unsure of their childhood immunisation status. Only two patients were on penicillin prophylaxis. Twenty-nine (15.9%) patients were on regular HU treatment. None of the patients was on chronic transfusion treatment, although the majority (n=119/182; 65.4%) had been transfused once or more in the past (mean lifetime transfusion 3.7 [SD 8.1]). None of the patients in our cohort had received stem cell therapy.

### Comparison of AI and HJB red cell counts across age groups between patients with SCD and controls

**Figure 1.**
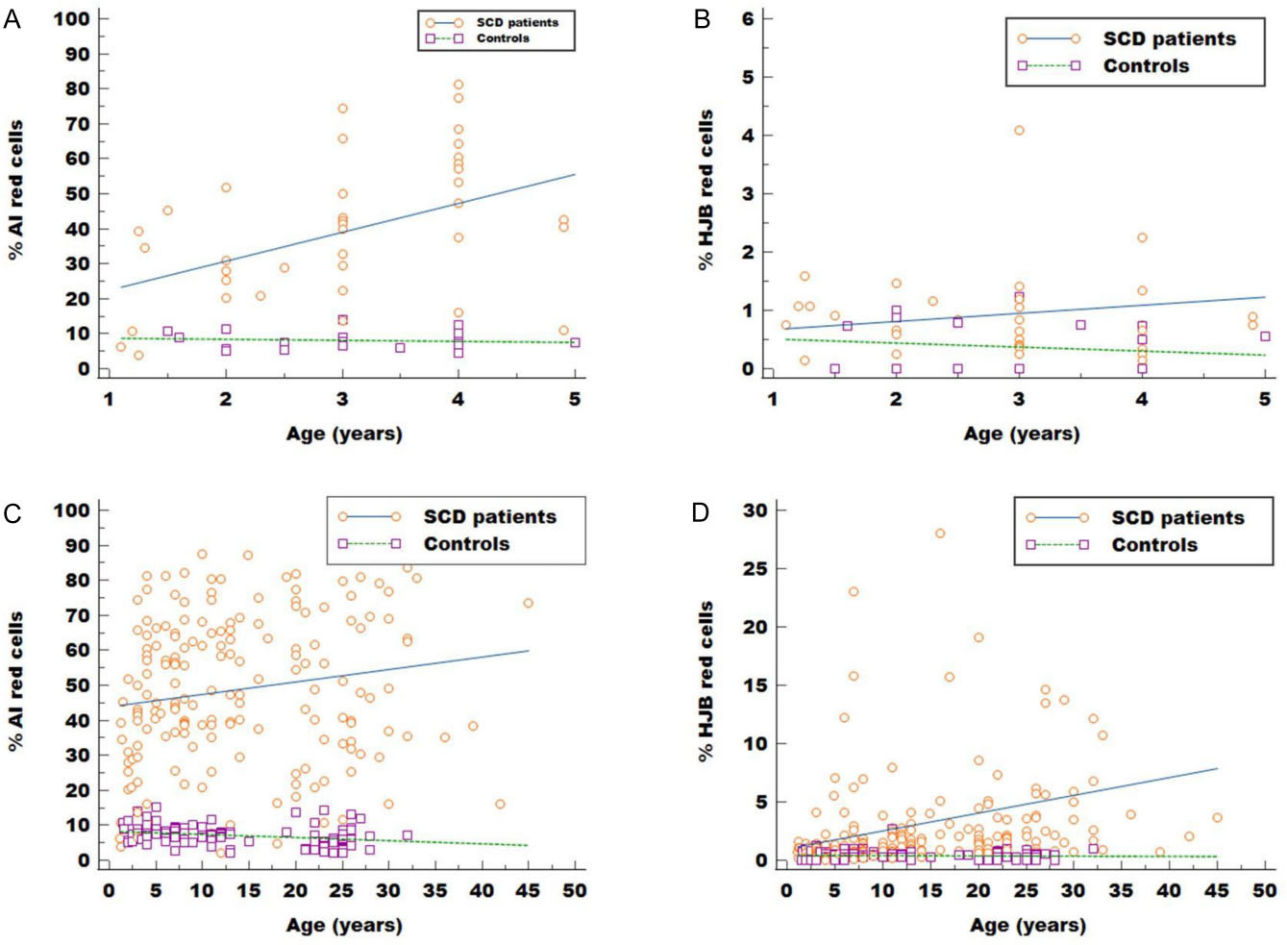
Scatter plots showing the relationship between age (*x*-axis) and the AI and HJB red cell counts (*y*-axis) among the study participants. (A) Among the group aged less than 5 y, the AI red cell counts were higher among the patients with SCD (n=37) and showed an increasing trend with age, whereas levels among the controls (n=22) were low and showed little variability with age. (B) Similarly, while the values of HJB increased steadily with age in the SCD group, there was an opposite trend among those aged less than 5 y in the controls. (C) Among the whole study population, the values of AI red cells were generally high across the SCD population with little variability across the age groups, whereas the values showed a progressive decline with age among the controls. (D) There was an increasing trend for HJB red cell counts with age among the patients with SCD; however, considerable variability was noted, as several younger children had elevated HJB red cells, while several older children had near-normal values. The levels of HJB red cells were generally low across all age groups of the controls.

Distribution of HJB and AI red cells showed consistently high levels of both markers in patients with SCD compared with controls across all age groups (Table 1). The progression of the frequencies of both markers with age differed between patients and controls (Figure 1A–D). Within the SCD population, the median percentage of HJB red cells rose steadily with increasing age, rising from 0.7% in children aged less than 5 y to 1.6% in those above 10 y and reaching 2.5% in those older than 15 y. The median percentage of AI red cells was lowest in children aged less than 5 y (median 40%) and rose to 50.0–57.5% in those aged over 5 y, including into adulthood. Within the control population, the opposite trend was noted. The AI red cell count decreased with increasing age from 7.5% in children aged less than 5 y to 5.6% in the adult group. The frequency of HJB red cells showed little variability with age among the controls.

**Table 1.** Comparison of AI and HJB red cell counts across age groups between patients with SCD and controls

	Patients with SCD (n=182)			Controls (n=102)			
Variable	N	Median (IQR)	Min–max	N	Median (IQR)	Min–max	p-value
%HJB RBCs							
<5 y	39	0.7 (0.4–1.1)	0.0–5.5	21	0.3 (0.0–0.7)	0.0–1.2	0.002*
5–9 y	37	0.9 (0.5–2.4)	0.1–23.0	21	0.5 (0.2–0.6)	0.0–0.9	0.001*
10–14 y	34	1.6 (0.9–2.5)	0.2–8.0	19	0.2 (0.2–0.5)	0.0–2.6	0.001*
≥15 y	72	2.5 (1.4–5.5)	0.4–28.2	41	0.2 (0.0–0.5)	0.0–0.9	0.001*
All	182	1.5 (0.7–3.1)	0.0–28.2	102	0.3 (0.1–0.5)	0.0–2.7	0.001*
%AI RBCs							
<5 y	39	40 (22–40)	3.7–81.2	21	7.5 (6.3–9.6)	4.5–13.9	0.001*
5–9 y	37	50 (40–50)	25.5–82.1	21	7.8 (6.3–9.1)	2.8–15.3	0.001*
10–14 y	34	58 (39–58)	2.0–87.5	19	7.2 (5.7–8.0)	2.0–11.3	0.001*
≥15 y	72	52 (34–50)	4.9–88.9	41	5.6 (3.6–8.3)	2.1–14.4	0.001*
All	182	47 (35–66)	2.0–88.9	102	7.1 (5.1–8.7)	2.0–15.2	0.001*

*Significant p-value by Mann–Whitney U test for within-age-group analysis. AI, argyrophilic inclusion; HJB, Howell–Jolly body.

### Distribution of AI and HJB red cell counts based on HU treatment and phenotype

The median age of patients with SCD on HU treatment was 11 y (IQR 16.6) and 12 y (IQR 16.0) in the non-HU group. The level of HbF was significantly higher among the patients with SCD on HU therapy (median 13.6%; IQR 9.2) compared with the non-HU group (median 8.1%; IQR 8.5) (p<0.001). However, levels of HJB red cells among the HU group were not significantly different compared with the non-HU group (median 1.2% vs 1.5%) (p=0.389). Similarly, levels of AI red cells were not significantly different between the HU group compared with the non-HU group (median 56.2% vs 46.1%) (p=0.789).

Among the SCD phenotype, the median HJB red cell counts were 1.4% (IQR 0.8–3.1) in patients with HbSS, 2.2% (IQR 1.2–3.1) in patients with HbSC and 1.2% in patients with HbSβ. The median AI red cell counts were 46.3% in patients with HbSS (IQR 35–66), 62.5% in patients with HbSC (IQR 27–47) and 35.2% in patients with HbSβ (IQR was not computed as there were only two patients). Further comparison based on phenotype was not performed because of the small number of patients with HbSC (n=5) and HbSβ (n=2). However, the mean age of the patients with HbSC (mean 20 y) and HbSβ (mean 25 y) appeared higher those of the patients with HbSS (mean 14 y).

### Association between AI and HJB red cell counts with clinical events and laboratory factors

To determine factors associated with the frequency of AI and HJB containing red cells, we analysed several clinical and laboratory parameters using univariate regression analysis (Table 2). None of the clinical events over the 12 months preceding the study including in-patient hospitalisation (r=0.034; p=0.650), febrile episodes (r=0.063; p=0.398) and painful crises (r=−0.044; p=0.558) was associated with the percentage of AI red cells. Similarly, the percentage of HJB red cells showed no association with in-patient hospitalisation (r=−0.010; p=0.894), febrile episodes (r=0.029; p=0.695) or painful crises (r=−0.185; p=0.252). The association of laboratory factors with the percentage of AI red cells was explored. Few factors retained significance in the final regression model (r=0.489; *r*^2^=0.239; adj. *r*^2^=0.223) (Table 3). The AI red cell counts associated positively with mean corpuscular hemoglobin (MCH) (β=0.321; p=0.0001) and negatively with HbF (β=−0.242; p=0.020). On exploration of the laboratory factors associated with frequency of HJB red cells, the final regression model (r=0.364, *r*^2^=0.132; adj. *r*^2^=0.118) showed a positive association with MCH (β=0.289; p=0.001) and a negative association with HbF (β=−0.259; p=0.002) (Table 3).

**Table 2.** Linear regression analysis for factors associated with AI and HJB red cell counts

		Univariate analysis for AI red cells		Univariate analysis for HJB red cells	
Variable	Mean (SD)	Standardised co-efficient β	p-value	Standardised co-efficient β	p-value
WBC (× 10^3^/µL)	13.8 (4.6)	0.071	0.344	0.045	0.545
Hb, g/dl	7.4 (1.7)	−0.185	0.013*	−0.234	0.002*
Platelets, count (× 10^6^/µL)	391 (166)	0.070	0.346	−0.025	0.741
MCV (fl)	82.8 (9.6)	0.237	0.001*	0.145	0.052
MCH (pg)	28.6 (3.7)	0.298	0.0001*	0.207	0.005*
MCHC (g/dl)	34.5 (1.8)	0.244	0.001*	0.188	0.011*
ANC (%)	5.8 (2.6)	0.153	0.041*	0.049	0.514
Reticulocyte (%)	8.3 (4.9)	0.235	0.001*	0.163	0.029*
Bilirubin total (umol/l)	35.1 (24.2)	0.100	0.205	0.248	0.001*
ASAT (IU/l)	21.0 (16.2)	0.137	0.081	0.007	0.931
HbF (%)	10.4 (7.1)	−0.258	0.001*	−0.202	0.010*
HbA_2_ (%)	3.3 (1.3)	0.065	0.468	0.152	0.088
HbS (%)	78.8 (9.6)	0.330	0.0001*	0.254	0.001*
Hospitalisation over last 12 months	0.51 (0.9)	−0.034	0.650	−0.010	0.894
Febrile episodes over last 12 months	2.4 (2.0)	0.063	0.398	0.029	0.695
Painful crises over last 12 months	2.4 (2.9)	−0.044	0.558	−0.185	0.252

WBC, white blood cells; MCV, mean corpuscular volume; MCH, mean corpuscular hemoglobin; MCHC, mean corpuscular hemoglobin concentration; ANC, absolute neutrophil count; ASAT, aspartate amino transferase; AI, argyrophilic inclusion; HJB, Howell–Jolly body. *All p-values were less than 0.05.

**Table 3.** Multivariate regression analysis for laboratory factors associated with HJB red cells

Variable	Co-efficient	Standard error	Standardised co-efficient β	p-value*
Dependent variable: AI red cells				
MCH (pg)	1.77	0.41	0.321	0.0001*
HbF (%)	−0.72	0.31	−0.242	0.020*
HbS (%)	0.41	0.23	0.184	0.073
Dependent variable: HJB red cells				
MCH (pg)	0.336	0.09	0.289	0.001*
HbF (%)	−0.160	0.05	−0.259	0.002*

MCH, mean corpuscular hemoglobin; AI, argyrophilic inclusion; HJB, Howell–Jolly bodies *All p-values were less than 0.05.

Variables consecutively removed from the model by the backward elimination:

AI red cells model: MCV, ANC, MCHC, reticulocyte count and hemoglobin.

HJB red cells model: HbS, bilirubin (total), reticulocyte count, MCHC and hemoglobin.

*Analysis was restricted to patients with SCD with complete clinical and laboratory data for the AI (n=146) and HJB red cell counts (n=129).

## Discussion

In the current study, levels of both AI and HJB red cells were significantly higher across all age groups of patients with SCD compared with controls, indicating the presence of splenic dysfunction among the patient group. This is in keeping with previous reports of raised AI^20^ and HJB red cell inclusions among patients with SCD.^4,21,22^ The effect of age produced different patterns on the two markers between the patients with SCD and controls. The frequency of red cell inclusions increased steadily with increasing age among the population with SCD; this was especially marked for the HJB red cell counts, corroborating previous studies from non-malaria regions.^2,4,23,24^ The findings are also similar to reports from studies that show increasing frequencies of red cell inclusions such as the pitted red cells with aging among patients with SCD.^8,9,22,25^ Our results suggest that splenic dysfunction begins early in life in SCD and continues to deteriorate into adulthood. Early loss of splenic function renders patients with SCD prone to infection^22,26^ and this has implications for clinical management, especially among children aged less than 5 y, which is the age associated with a high risk for bacterial infections with encapsulated organisms (namely *Neisseria meningitidis, Streptococcus pneumoniae* and *Haemophilus influenzae type b*). Management strategies are aimed at preventing severe sepsis and its related morbidity; this includes patient education, prompt and aggressive treatment of fever, vaccination prophylaxis and antibiotic chemoprophylaxis, as recommended for all individuals with impaired splenic function at risk of fulminant infection.^15^ Splenic dysfunction also results in poor clearance of malaria-infected red cells,^27^ thus malaria preventive measures, such as use of insecticide-treated bed nets and malaria chemoprophylaxis according to national guidelines, are recommended in patients with SCD who are residing in malaria endemic countries.^28^

The levels of both markers were also high among patients with compound heterozygotes for SCD in this study; because of the small number of this group (HbSC, n=5 and HbSβ, n=2), we could not analyse the effect of phenotype on both markers of splenic dysfunction. However, the observed raised AI and HJB red cell counts among the patients with HbSC in this study contrast with findings from previous studies of preserved splenic function among patients with HbSC.^23,29^ The older age of the patients with HbSC (mean 20 y; range 9–45 y) in the current study may account for this observation. The influence of age on both markers produced an opposite trend in the control group compared with the patients with SCD. In the controls, the AI red cell count declined with increasing age; this may be explained by the progressive and physiological increase in spleen size of normal individuals,^30^ and thus the ability to more effectively clear out inclusions. The HJB red cell counts in controls were not affected by increasing age; the median value remained stable across all age groups (median 0.3%; p=0.834). HJBs are formed at a very low frequency and are rarely encountered in normal individuals because of the high efficiency of removal by the healthy spleen.^31^ Our findings are similar to previous reports of HJBs estimated by flow cytometry in normal individuals.^32,33^

We also sought to determine laboratory factors associated with the HJB and AI red cell inclusions. The frequencies of both AI and HJB red cells showed a significant positive association with MCH and a negative association with HbF levels. This suggests that both markers may be influenced by the same process in the spleen. We noted that both markers correlated more strongly with the MCH (p=0.001) than with HbF (AI count, p=0.020; HJB count, p=0.002), indicating that MCH may be the more important association with splenic dysfunction than HbF among our patient population. It is not known whether the observed association with MCH is due to the presence of the alpha thalassemia trait among our population with SCD, which is a condition known to ameliorate splenic dysfunction.^6,7,9,34,35^ Alpha thalassemia is highly prevalent (36–54%) among individuals of West and East African origin due to its protective effect on malaria.^36–38^ The MCH is a measure of the average amount of hemoglobin in the red cells; a high level favors polymerisation of HbS, thereby inducing intravascular sickling of the red cells.^39^ The splenic environment also favors the process of red cells sickling; the blood within the splenic cords has a high hematocrit and stagnation of red cells within the cords leads to hypoxia and acidosis, all of which promote red cell sickling.^40^ Therefore, a combination of a splenic hostile environment and high MCH is likely to favor the sickling process in patients with SCD and to result in accelerated splenic dysfunction.

The negative association of HbF level with increased frequencies of AI and HJB red cell inclusions in our study is in keeping with the influence of HbF in preserving spleen function. High levels of HbF are associated with reduced intracellular HbS polymerisation, and hence preservation of spleen function in patients with SCD as evidenced in reports from the USA^3,10,22^ and India.^9^ Normal or near-normal spleen function has also been reported in the majority of patients with SCD with high HbF from the Middle East.^7,41–43^ The protective effect of HbF on splenic function has, however, not been confirmed in all studies.^6,25^ We found that a high level of HbS was associated with increased AI red cell counts. The high level of HbS favors the process of polymerisation and ultimately impacts the frequency of intravascular sickling of red cells, which worsens spleen function.

None of the clinical parameters analysed in the current study showed an association with both markers of splenic dysfunction. Nevertheless, the majority patients with SCD reported having at least one episode of fever over the preceding year and about a third required hospitalisation; thus, it is reasonable to recommend antipneumococcal prophylaxis and antimalaria prophylaxis especially for those less than 5 y old. Only a few of the patients with SCD (15.9%) were on HU treatment in the current study, this may have affected our ability to detect any significant association with both markers of splenic dysfunction. However, as expected, a higher HbF among the patients with SCD on HU treatment compared with the non-HU group was noted. HU induces production of HbF and has been used as a disease modifying agent in SCD over the past three decades.^3^ Previous studies have shown that an increase in the level of HbF was associated with splenic preservation^10,44^; other studies have not observed any improvement in splenic function among their HU-treated patients.^45,46^ Despite the disparity in reports, given the progressive increase noted with age in the markers of splenic dysfunction in this study, starting HU treatment early may be beneficial, as the associated increase in HbF levels may allow the maintenance or restoration of splenic function before irreversible fibrosis and loss of function occur.

## Limitations

The cross-sectional design of the study meant we could not monitor longitudinally the changing levels of markers of splenic dysfunction with age and therefore identify the exact onset of splenic dysfunction among our patients. However, the paired assessment of markers of splenic dysfunction between comparable age groups of SCD and healthy controls enabled us to document the changing pattern across a broad spectrum of age.

### Conclusions

An age-related increase in markers of splenic dysfunction (i.e. HJB and AI red cell inclusions) occurred among our patients with SCD. Although both markers of hyposplenism increased with increasing age among the patients with SCD in our study, there was less variability after age 5 y with the AI red cell counts. The steady increase with age observed in the HJB red cell counts indicates that this would be the preferred method to track splenic function in each individual. We observed a negative association between HbF and levels of both markers of splenic dysfunction. Although we found no association between HU therapy and both markers, possibly due to the small number of patients on HU, the high HbF among patients on HU and the known protective effect of HbF on splenic function suggest that early administration of HU may be beneficial in ameliorating the course of splenic dysfunction among patients with SCD.

## Data Availability

The data underlying this article are available in the manuscript, figures and tables. Further request can be addressed to the corresponding author.
